# Morphological and biochemical responses of tropical seagrasses (Family: Hydrocharitaceae) under colonization of the macroalgae *Ulva reticulata* Forsskål

**DOI:** 10.7717/peerj.12821

**Published:** 2022-01-18

**Authors:** Lau Sheng Hann Emmclan, Muta Harah Zakaria, Shiamala Devi Ramaiya, Ikhsan Natrah, Japar Sidik Bujang

**Affiliations:** 1 Laboratory of Marine Biotechnology, Institute of Bioscience, Universiti Putra Malaysia, UPM Serdang, Selangor Darul Ehsan, Malaysia; 2 Department of Aquaculture, Faculty of Agriculture, Universiti Putra Malaysia, UPM Serdang, Selangor Darul Ehsan, Malaysia; 3 Department of Crop Science, Faculty of Agriculture Science and Forestry, Universiti Putra Malaysia, Bintulu, Sarawak, Malaysia; 4 Department of Biology, Faculty of Science, Universiti Putra Malaysia, UPM Serdang, Selangor Darul Ehsan, Malaysia

**Keywords:** Seagrass, Macroalgal bloom, Hydrocharitaceae, Morphometric, Antioxidant activity, Land reclamation

## Abstract

**Background:**

Coastal land development has deteriorated the habitat and water quality for seagrass growth and causes the proliferation of opportunist macroalgae that can potentially affect them physically and biochemically. The present study investigates the morphological and biochemical responses of seagrass from the Hydrocharitaceae family under the macroalgal bloom of *Ulva reticulata*, induced by land reclamation activities for constructing artificial islands.

**Methods:**

Five seagrass species, *Enhalus acoroides*, *Thalassia hemprichii*, *Halophila ovalis*, *Halophila major,* and *Halophila spinulosa* were collected at an *Ulva reticulata*-colonized site (MA) shoal and a non-*Ulva reticulata*-colonized site (MC) shoal at Sungai Pulai estuary, Johor, Malaysia. Morphometry of shoots comprising leaf length (LL), leaf width (LW), leaf sheath length (LSL), leaflet length (LTL), leaflet width (LTW), petiole length (PL), space between intra-marginal veins (IV) of leaf, cross vein angle (CVA) of leaf, number of the cross vein (NOC), number of the leaf (NOL) and number of the leaflet (NOLT) were measured on fresh seagrass specimens. Moreover, *in-situ* water quality and water nutrient content were also recorded. Seagrass extracts in methanol were assessed for total phenolic content (TPC), total flavonoid content (TFC), 2,2-diphenyl-1-picrylhydrazyl radical scavenging activity (DPPH), 2,2′-azino-bis(3-ethylbenzthiazoline-6-sulfonic acid radical cation scavenging activity (ABTS), and ferric reducing antioxidant power (FRAP).

**Results:**

Seagrasses in the *U. reticulata*-colonized site (MA) had significantly higher (*t*-test, *p* < 0.05) leaf dimensions compared to those at the non-*U. reticulata* colonized site (MC). Simple broad-leaved seagrass of *H. major* and *H. ovalis* were highly sensitive to the colonization of *U. reticulata*, which resulted in higher morphometric variation (*t*-test, *p* < 0.05) including LL, PL, LW, and IV. Concerning the biochemical properties, all the seagrasses at MA recorded significantly higher (*t*-test, *p* < 0.05) TPC, TFC, and ABTS and lower DPPH and FRAP activities compared to those at MC. Hydrocharitaceae seagrass experience positive changes in leaf morphology features and metabolite contents when shaded by *U. reticulata*. Researching the synergistic effect of anthropogenic nutrient loads on the interaction between seagrasses and macroalgae can provide valuable information to decrease the negative effect of macroalgae blooms on seagrasses in the tropical meadow.

## Introduction

Seagrasses are marine angiosperms growing from shallow coastal to a depth of about 70 m (Japar Sidik & Muta Harah, 2003; Waycott et al., 2009). They form extensive meadows, composed of a single or a mixture of species (Japar Sidik, Muta Harah & Arshad, 2006), and are one of the most productive ecosystems in the world (Larkum, Orth & Duarte, 2006). The meadows are of ecological importance since they function as habitat and food source to diverse communities of aquatic organisms (Japar Sidik, Muta Harah & Arshad, 2006), as a stabilizer of sediments from erosion (Waycott et al., 2009; Potouroglou et al., 2017) and as a nutrient cycle regulator (Hemminga & Duarte, 2000). However, due to natural and anthropogenic problems that occur persistently in coastal areas, seagrass meadows have been declining worldwide (Orth et al., 2006; Waycott et al., 2009; Short et al., 2011; Schmidt et al., 2012; Japar Sidik, Muta Harah & Short, 2018).

Rapid human population growth has increased the land demand for housing areas and has subsequently led to the extension of artificial shorelines through reclamation activity (Chee et al., 2017). The rapid and large-scale reclamation has resulted in the disappearance of gulfs and bays, increased water pollution, and frequent harmful algal blooms (Liu & Su, 2015). Such effects have surfaced in several coastal waters of Malaysia, including Merambong shoal, an important seagrass bed located at Sungai Pulai estuary Johor, Malaysia (Japar Sidik, Muta Harah & Arshad, 2014). The reclamation has changed the surrounding water quality, caused eutrophication, and led to the mass proliferation of the macroalgae, *U. reticulata* Forsskål (Japar Sidik, Muta Harah & Arshad, 2014). Coastal eutrophication remains as one of the major anthropogenic inputs that can severely impact seagrass beds’ ecosystem structure and services (Burkholder, Tomasko & Touchette, 2007; Van Katwijk et al., 2010; Schmidt et al., 2012; Han & Liu, 2014; Nakaoka et al., 2014). Under normal circumstances, nutrient enrichment enhances the growth and production of seagrasses in oligotrophic water (Hughes et al., 2004), but an excessive nitrogen enrichment above the optimal range can disrupt their growth and lead to seagrass dying off (Schmidt et al., 2012). Heavy use of agricultural fertilizers, intense burning of fossil fuels, and uncontrolled wastewater discharges are some of the human activities that contribute to anthropogenic nitrogen loading of the ocean (Taylor et al., 1995; Vitousek et al., 1997). Nitrogen loading causes a shift in the main primary producer composition in the meadows, from seagrass to phytoplankton and fast-growing macroalgae (Han & Liu, 2014).

Macroalgae such as *Ulva* sp. have a broad tolerance range toward nitrogen and phosphate concentrations as long as the light is not a limiting factor (Luo, Liu & Xu, 2012; Liu et al., 2013). The rapid growth and proliferation of some macroalgae, *e.g., Caulerpa* sp., *Cladophora* sp., and *Ulva* sp., coupled with nutrient enrichment, have been documented in several tropical and temperate bioregions of seagrasses (Han & Liu, 2014). Such phenomenon has significantly reduced the light availability and promotes the development of anoxic conditions in seagrass beds (Cabaço et al., 2013; Han & Liu, 2014), and as a result, the changes have greatly affected the seagrass morphology (Fitzpatrick & Kirkman, 1995; Longstaff et al., 1999; La Nafie et al., 2013; York et al., 2013) and biochemical properties (Cuny et al., 1995; Agostini, Desjobert & Pergent, 1998; Ferrat, Fernandez & Dumay, 2001; Dumay et al., 2004). Eutrophic water creates turbid and anoxic conditions coupled with an algal bloom that can negatively affect the seagrass’ shoot density and biomass (Cabaço et al., 2013), while a reduction in light causes the seagrass unable to maintain positive carbon balance and hinder their growth (Ralph et al., 2007). Linear-leaved seagrasses have reduced shoot density, shoot biomass, and leaf growth rate when cultured under low light conditions, *e.g*., in *Posidonia australis* (Fitzpatrick & Kirkman, 1995) and *Zostera muelleri* (York et al., 2013). In comparison, small-leaved seagrasses such as *Halophila* species are more sensitive to prolonged light deprivation. The rapid die-off of the *H. ovalis* after 30 days under a low light regime was reported due to a shortage of carbohydrates and phytotoxic build-up in their thin leaves and small below-ground biomass (Longstaff et al., 1999).

Apart from morphological changes, seagrasses actively synthesize secondary metabolites in response to environmental disturbances (Grignon-Dubois et al., 2012; Subhashini et al., 2013). Phenolic compounds are examples of secondary metabolites abundant in seagrasses (Vergeer, Aarts & De Groot, 1995; Vergeer & Develi, 1997; Ferrat, Fernandez & Dumay, 2001; Dumay et al., 2004). These metabolites are essential for regular biological activity and protection against environmental stressors associated with their habitat (Subhashini et al., 2013), and are affected by seasonal changes (Ferrat, Fernandez & Dumay, 2001; Dumay et al., 2004; Achamlale, Rezzonico & Grignon-Dubois, 2009; Steele & Valentine, 2012), light intensity (Vergeer, Aarts & De Groot, 1995), temperature (Vergeer, Aarts & De Groot, 1995), pH (Arnold et al., 2012), nutrient loading (Cannac et al., 2006), heavy metal pollution (Ferrat et al., 2012), pathogen infestation (Vergeer & Develi, 1997), grazing activity (Steele & Valentine, 2012) and macroalgal bloom (Dumay et al., 2004). Temperate seagrasses are reported to have high phenolic content during spring and summer, attributed to the higher temperature and more prolonged sunlight exposure, in contrast to during autumn and winter (Ferrat, Fernandez & Dumay, 2001; Dumay et al., 2004; Achamlale, Rezzonico & Grignon-Dubois, 2009; Steele & Valentine, 2012). High light intensity enhances photosynthetic activity in seagrasses and triggers the higher accumulation of phytochemicals in the plants (Vergeer, Aarts & De Groot, 1995). However, increasing temperature beyond the optimal growth level of seagrasses (Vergeer, Aarts & De Groot, 1995) and acidified seas (Arnold et al., 2012), can disrupt the biosynthesis of the metabolite, which would lead to a noticeable reduction of total phenolic acids in seagrasses. Inshore fish farming nutrient loading, *i.e.*, ammonia and phosphate, have been shown to stimulate the production of total proanthocyanidins, total, and simple flavonols in *P. oceanica* (Cannac et al., 2006). Seagrasses found near polluted industrial sites possessed lower phenolic content due to a high concentration of heavy metal accumulated in plant cells that inhibit their biosynthesis (Ferrat et al., 2012). The role of secondary metabolites as a defense mechanism in seagrass is still inadequately investigated (Heck & Valentine, 2006), and there is some evidence suggesting that infestation of *Labyrinthula* sp. on seagrass triggers higher production of phenolic acids (Vergeer & Develi, 1997; Steele et al., 2005). According to Steele & Valentine (2012), seagrass frequently grazed by aquatic organisms develop higher phenolic acid content and several tannins as a deterrent from an herbivore. Colonization of epiphytic macroalgae did not greatly influence the overall phenolic production on temperate seagrasses; however, there was still apparent changes in particular phenolic compounds and number of tannin cells (Cuny et al., 1995; Agostini, Desjobert & Pergent, 1998; Ferrat, Fernandez & Dumay, 2001; Dumay et al., 2004). The phenolic increment appears to be at a species-specific interaction, as observed during the colonization of macroalgae *Caulerpa taxifolia* on *P. oceanica*, not for *Caulerpa racemosa* (Dumay et al., 2004). To date, most studies have focused on the effects of abiotic and biotic environmental factors on temperate seagrass responses, and similar studies are still lacking for tropical seagrass. Morphological and biochemical responses of the tropical seagrasses under macroalgal bloom can vary from temperate seagrasses. Frequent occurrence of coastal macroalgal bloom in seagrass meadow has also prompted investigation of its impact on seagrass survival and adaptability. Therefore, the present study investigates whether the mass proliferation of *U. reticulata* can cause an adverse effect on the morphology and metabolite content of the Hydrocharitaceae seagrass of *E. acoroides*, *T. hemprichii*, *H. ovalis*, *H. major*, and *H. spinulosa*.

## Materials & Methods

### Seagrass meadow: observation and sample collection

**Figure 1:**
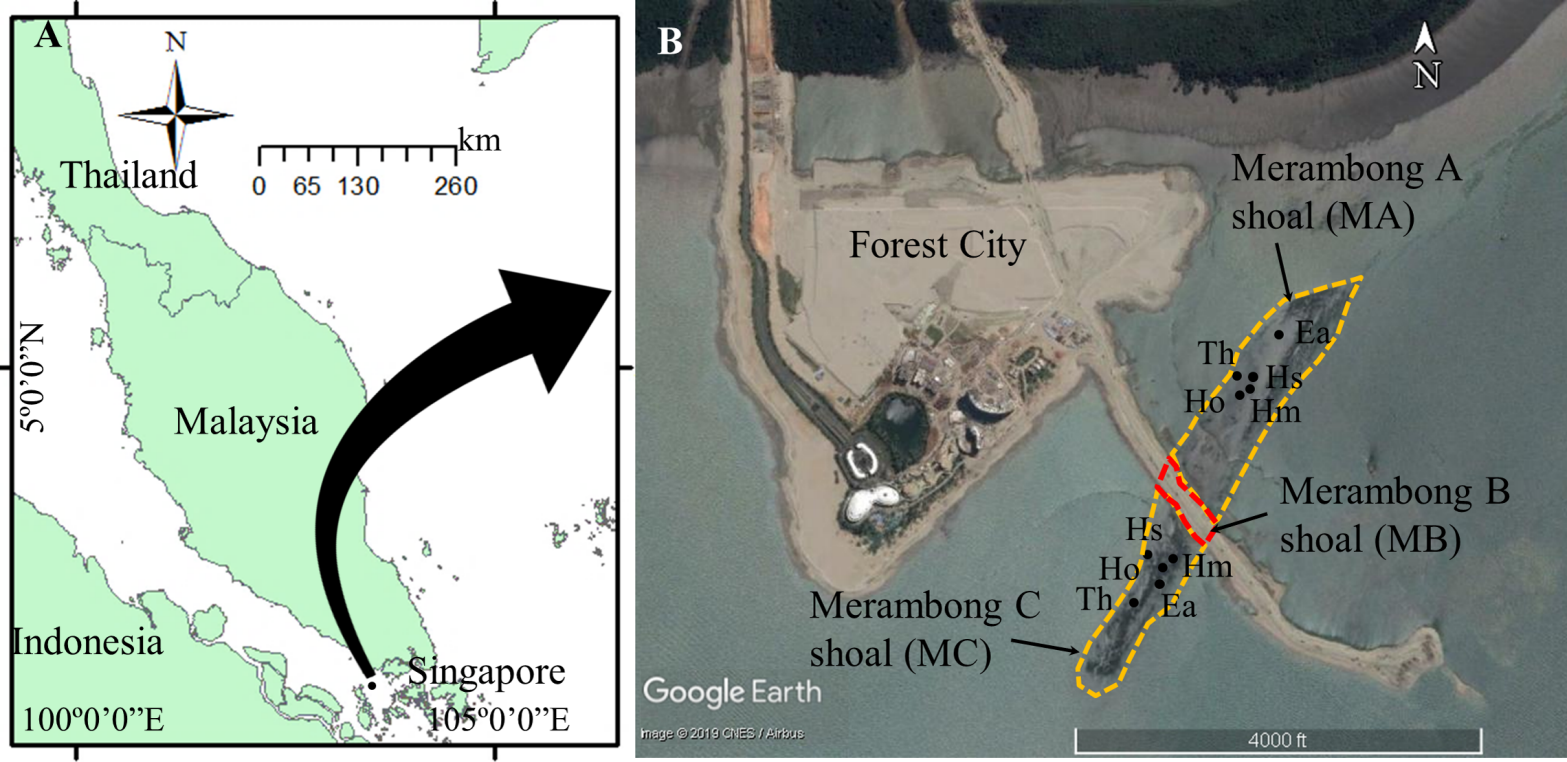
Seagrass collection sites at Merambong shoal, Sungai Pulai Estuary, Johor, Malaysia. Map Source: (A) ArcGIS 10.3 for Desktop and (B) Google Earth Image ©2019 CNES/Airbus with global positioning system (GPS) coordinates according to seagrass species at Merambong A and Merambong C shoals. Ea, *Enhalus acoroides*; Th, *Thalassia hemprichii*; Hm, *Halophila major*; Ho, *Halophila ovalis*; and Hs, *Halophila spinulosa*.

**Figure 2:**
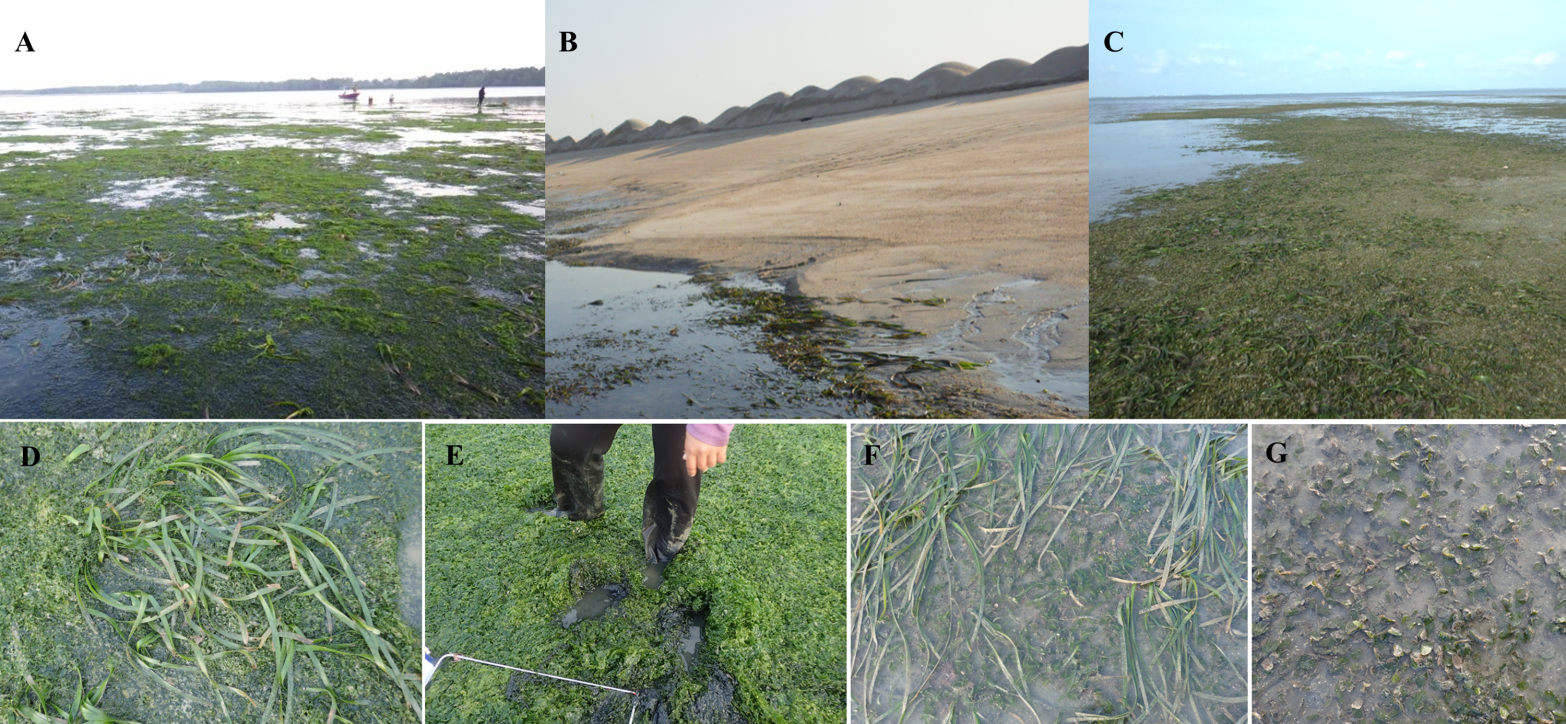
Photos of Merambong shoal conditions during the land reclamation activities. (A) Merambong A, (B) Merambong B, (C) Merambong C. (D) *Enhalus acoroides* long leaf still noticeable above the dense cover of *Ulva reticulata* on Merambong A shoal, and (E) the macroalgae decomposed and formed black sediment underneath. (F & G) Seagrasses in Merambong C shoal are growing abundantly and recovering from the land reclamation activities.

Seagrasses were collected at an intertidal Merambong shoal, Sungai Pulai estuary, Johor, on 15 and 16 July 2014 during low spring tides. The shoal is exposed to air for 1–2 h during low tide and completely submerged during high tide (3–4 m depth). Through a coastal land reclamation project initiated in January 2014 along the coastline of Sungai Pulai estuary, Johor has successfully built up a sand-filled embankment from the main island (Forest City) across the middle part of Merambong shoal, separated into three main parts: (1) Merambong A (MA, facing toward Malaysia-Singapore second link expressway), (2) Merambong B (MB, sand-filled embankment), and (3) Merambong C (MC, facing toward the Port of Tanjung Pelepas) (Figs. 1 and 2). The seagrass bed comprises eight seagrass species (*Enhalus acoroides* (L.f.) Royle, *Thalassia hemprichii* (Ehrenb. ex Solms) Asch*.*, *Halophila major* (Zoll.) Miquel, *Halophila ovalis* (R.Br.) Hook.f., *Halophila spinulosa* (R.Br.) Asch., *Cymodocea serrulata* (R. Br.) Aschers. & Magnus, *Halodule pinifolia* (Miki) den Hartog and *Halodule uninervis* (Forssk.) Aschers*.*) has been greatly reduced to 21.1 ha in 2014 (Japar Sidik, Muta Harah & Short, 2018) from 26.3 ha in 2013 (Muta Harah et al., 2014). The embankment has restricted the water flow and promotes the proliferation of opportunistic macroalgae *U. reticulata* in Merambong A. As a result, nearly 90% of the seagrasses in Merambong A were covered with the extensive and thick (5–30 cm) layer of *U. reticulata* (MA, *U. reticulata*-colonized site). The presence of the macroalgae was also detected in Merambong C, but in a small quantity (estimated <1% cover), serving as the non-colonized site (MC, non*-U. reticulata*-colonized site). Water quality parameters, *i.e.*, temperature, atmospheric pressure, dissolved oxygen, conductivity, total dissolved solids, salinity, and pH, surrounding the shoals were measured using a YSI professional plus handheld multi-parameter instrument (YSI Inc., USA, Table 1). Water visibility was measured using a 30 cm diameter Secchi disc (Abal & Dennison, 1996). Total suspended solids (TSS) were determined in the laboratory, according to EPA Method 160.2 (United States Environmental Protection Agency, 1999). Water ammonia (NH_3_^−^), nitrate (NO_3_^−^), nitrite (NO_2_^−^), and orthophosphate (PO_4_^3−^) contents were determined using a Hach DR/2400 Spectrophotometer according to Hach DR/2400 Spectrophotometer Procedure Manual (Hach Company, 2004).

**Table 1:** Water quality parameters given as mean ±standard deviation and ranges of Merambong A and C shoals.

**Parameter**	**MA**	**MC**	**Mean diff.**	**t**	**df**	**p**	**Status compared to MMWQS (Class 1)**
Temperature (°C)	30.13 ± 0.37 (29.80–30.60)	30.05 ± 0.27 (29.80–30.30)	0.08	0.44	10	0.67	Values slightly higher than the criteria set limit of ≤2 °C increase over max. ambient.
Atmospheric pressure (mmHg)	757.55 ± 0.21 (757.30–757.80)	757.53 ± 0.19 (757.30–757.70)	0.02	0.15	10	0.89	Not stated.
Dissolved oxygen (mg L^−1^)	5.16 ± 0.67 (4.70–6.52)	5.23 ± 0.30 (4.95–5.71)	−0.07	−0.23	10	0.82	Min. values MA, MC and max. value MC are below criteria set limit of >6.0 mg/L.
Conductivity (µS cm^−1^)	52006 ± 1026 (50879–52948)	52956 ± 103 (52793–53093)	−950.33	−2.26	10	0.05	Not stated.
Total dissolve solid (mg L^−1^)	30788 ± 789 (29900–31525)	31395 ± 130 (31200–31525)	−606.67	−1.86	10	0.09	Not stated.
Salinity (PSU)	30.68 ± 0.87 (29.71–31.47)	31.34 ± 0.15 (31.11–31.47)	−0.66	−1.82	10	0.10	Not stated.
pH	7.40 ± 0.09 (7.24–7.48)	7.41 ± 0.09 (7.24–7.51)	−0.01	−0.10	10	0.93	Min. and max. values within the criteria set limit of 6.5-9.0.
Total suspended solid (mg L^−1^)	102.33 ± 7.43 (94.00–113.60)	91.60 ± 14.59 (69.20–104.00)	10.73	1.61	10	0.14	Min. and max. values were above 25 mg/L under Class 1 (Sensitive Marine Habitats).
Visibility (m) Secchi disc depth (m)	0.65 ± 0.22 (0.40–1.00)	0.78 ± 0.17 (0.50–1.00)	−0.13	−1.18	10	0.27	Not stated.
Ammonia (mg L^−1^)	0.06 ± 0.02 (0.03–0.08)	0.03 ± 0.02 (0.00–0.04)	0.03	3.23	10	**0.01**	Min. and max. levels below the criteria set limit of 35.0 mg/L.
Nitrate (mg L^−1^)	1.88 ± 0.73 (0.90–2.70)	0.97 ± 0.50 (0.40–1.80)	0.92	2.55	10	**0.03**	Min. and max. values below the criteria set limit of 10.0 mg/L.
Nitrite (mg L^−1^)	0.013 ± 0.007 (0.004–0.021)	0.009 ± 0.006 (0.001–0.016)	0.01	1.17	10	0.27	Not stated.
Phosphate (mg L^−1^)	1.33 ± 0.88 (0.47–2.46)	0.83 ± 0.97 (0.11–2.26)	0.50	0.93	10	0.37	Min. and max. levels below the criteria set limit of 5.0 mg/L.

**Notes.**

Mean ± standard deviation in a row with bold *p*<0.05 is significantly different (*t*-test). The range of minimum and maximum values, are recorded and given in the parentheses. The listed Malaysian Marine Water Quality Standards (MMWQS) recommended water quality limits for Sensitive Marine Habitats include seagrass (Class 1). MA: Merambong A shoal and MC: Merambong C shoal.

Approximately 100 g of fresh leaves of *E. acoroides*, *T. hemprichii*, *H. major, H. ovalis*, and *H. spinulosa* were manually collected. Photos and GPS coordinates (latitude and longitude) of seagrass species were recorded using an underwater camera Ricoh WG-4 GPS (Ricoh, Japan) as depicted in Fig. 1 and Table 1. Samples were cleaned of adhering materials, stored in zip-lock plastic bags, and kept in an ice chest before transporting to the laboratory. Seagrasses collected were divided into two portions; (i) morphological measurement and (ii) biochemical analyses.

### Morphological observation

Fresh seagrass samples and only the oldest undamaged leaves on a leafy shoot (the third leaf and above for linear leaves seagrass) were measured. For linear leaves, *E. acoroides* and *T. hemprichii*, leaf length (LL), leaf width (LW), leaf sheath length (LSL), and the number of leaves (NOL) were recorded. For simple broad leaves, *H. major* and *H. ovalis,* leaf length (LL), leaf width (LW), leaf sheath length (LSL), petiole length (PL), space between intra-marginal veins (IV), cross vein angle (CVA) and the number of cross vein (NOC) were documented. For broad compound leaves, *H. spinulosa*, leaf length (LL), leaf width (LW), leaflet length (LTL), leaflet width (LTW), and the number of leaflets (NOLT) were recorded. The seagrass LL, LW, LSL, PL, LTL, LTW, and IV were measured using a digital vernier caliper (Mitutoyo, Japan). CVA was measured using a protractor while observing the leaves under Leica ES2 stereomicroscope (Leica Microsystems Pte Ltd, Singapore). The NOL and NOLT were counted based on per shoot. Leaf length to leaf width (LL:LW) and leaf length to leaf sheath length (LL:LSL), leaf length to petiole length (LL:PL), leaflet length to width (LTL:LTW), and half lamina width to intra-marginal vein ($ \frac{1}{2} $LW:IV) ratios were derived from the data collected.

### Seagrass extract preparation

The leaves of the seagrass were separated from the whole plant. Any debris or attached epiphytes were removed from the leaves and then cleaned and rinsed with distilled water. The cleaned leaves were placed into an aluminum foil sheet and oven-dried at 40 °C in an air-circulating Memmert UF160 oven (Memmert, Germany) for three days until dry. The dried leaves were then ground into a fine powder using a blender (Panasonic, Japan), and were sifted through a 250 µm laboratory test sieve (ABSS, Australia). The fine powders <250 µm were used for the extraction following Kannan, Arumugam & Anantharaman (2010). One gram of the powdered material was extracted with 10 mL of absolute methanol solvent on an orbital shaker Protech model 719 (Protech, Malaysia) for 24 h. The mixtures were filtered through Whatman filter paper no. 1 (Sigma-Aldrich, USA), and the residues retained on the filter paper were rinsed thrice with five mL absolute methanol. The collected filtrates were pooled and then filtered twice with new filter paper. The filtrates were vacuum-dried at 55 °C and 200–250 mbar in a rotary vacuum evaporator IKA^®^RV 10 (IKA, Germany).

### Total phenolic content (TPC)

The phenolic content of the methanolic seagrass extract was determined using Folin-Ciocalteu assay (Zhang et al., 2006). Before the test, 5 mg mL^−1^ concentrations of each seagrass extract were prepared in absolute methanol. Twenty microliters (20 µL) of seagrass extract, gallic acid (concentration ranging from 0.0–0.4 mg mL^−1^), and absolute methanol (blank) were placed in separate wells in a microplate 96-well using a 10–100 µL micropipette (Eppendorf, Germany). Then, 100 µL of 2N Folin-Ciocalteau phenol reagent was dispensed into the wells using a 12-channel micropipette (Eppendorf, Germany). The samples were mixed and incubated for 5 min at room temperature. After incubation, 80 µL of 7.5% sodium carbonate was added, and the reaction mixture was kept in the dark condition for 30 min at room temperature. The reaction mixture absorbance (extract, gallic acid, and blank) was measured at 765 nm wavelength using a Thermo Scientific Multiskan FC microplate photometer (Thermo Scientific, USA). The calibration curve of gallic acid’s concentration versus absorbance was plotted, and the total phenolic content was calculated from the curve equation as milligram gallic acid equivalent per gram dry weight of extract (mg GAE g^−1^ extract).

### Total flavonoid content (TFC)

The flavonoid content of the extracts was estimated using aluminum nitrate colorimetric assay (Herald, Gadgil & Tilley, 2012). Similar to TPC, each extract was prepared to a 5 mg mL^−1^ concentration. Thirty microliters (30 µL) of the extract, quercetin (concentration ranging from 0.0–0.4 5 mg mL^−1^), and methanol (blank) were dispensed into separate wells in a 96-well microplate. The samples were mixed with 40 µL of 10% aluminum nitrate and further diluted with 180 µL of deionized water. After 10 min incubation, the reaction mixture absorbance (extract, quercetin, and blank) was measured at 269 nm wavelength using a microplate photometer. The standard curve of quercetin’s concentration versus absorbance was plotted. The total flavonoid content was calculated from the curve equation as milligram quercetin equivalent per gram dry weight of extract (mg QE g^−1^ extract).

### DPPH radical scavenging activity

The antioxidant activities were measured using a 2,2-diphenyl-1-picrylhydrazyl (DPPH) assay following the method described by Cheng, Moore & Yu (2006). Before the test, each extract was diluted with methanol to a concentration of 150 µg mL^−1^. One hundred microliter (100 µL) of the seagrass extract, 6-Hydroxy-2,5,7,8-tetramethylchromane-2-carboxylic acid (Trolox, concentration ranging from 0.02 - 0.09 mM), and methanol (blank) were dispensed into separate wells in a 96-well microplate. An equal amount of 0.16 mM DPPH prepared in methanol was then added into the wells using a 12-channel micropipette. The microplate was kept under dark condition at room temperature of 24 °C for 40 min. The reaction mixture absorbance (extract, Trolox, and blank) was measured at 515 nm wavelength using a microplate photometer. The standard curve of trolox’s concentration versus absorbance was plotted. The DPPH quenching activity was expressed from the curve equation as micromolar Trolox equivalent per gram dry weight of extract (µM TE g^−1^ extract).

### ABTS radical cation scavenging activity

Antioxidant activities of the extracts were also determined using 2,2′-azino-bis(3-ethylbenzthiazoline-6-sulfonic acid (ABTS) assay (Re et al., 1999). Briefly, the ABTS radical was generated by mixing 7 mM ABTS with 2.45 mM potassium persulfate in a 2:1 ratio, respectively. The mixture was incubated at room temperature for 12 h before use. The absorbance value of the ABTS radical solution was measured at 734 nm wavelength using a microplate photometer and was adjusted to 0.70 ± 0.01 absorbance value by dilution with deionized water. Ten microliters (10 µL) of the extract (150 µg mL^−1^), Trolox (concentration ranging from 0.02–0.09 mM), and methanol (blank) were dispensed into separate wells in a 96-well microplate. 190 µL of the prepared ABTS radical solution were then dispensed into the well and incubated under dark conditions for 10 min. The reaction mixture absorbance (extract, Trolox, and blank) was measured at 734 nm wavelength. The standard curve of trolox’s concentration versus absorbance is plotted. The ABTS scavenging activity was expressed from the curve equation as micromolar Trolox equivalent per gram dry weight of extract (µM TE g^−1^ extract).

### Ferric reducing antioxidant power (FRAP)

The antioxidant reducing potential of seagrass extracts was measured colorimetrically based on the formation of colored ferrous tripyridyltriazine complex from ferric solution (Benzie & Strain, 1996). The FRAP reagent was prepared by mixing 300 mM acetate buffer (pH 3.6), 10 mM of 2,4,6-Tri(2-pyridyl)-s-triazine in HCl, and 20 mM iron(III) chloride in a ratio of 10:1:1. Ten microliter (10 µL) of the extract (150 µg mL^−1^), iron(II) sulfate (concentration ranging from 0.28 × 10^−4^–2.22 ×10^−4^ mg) and methanol (blank) were dispensed into separate wells in a 96-well microplate. Then, 200 µL of the prepared FRAP solution were dispensed into the well and incubated under dark conditions for 10 min. The reaction mixture absorbance (extract, iron(II) sulfate, and blank) was measured at 593 nm wavelength. The standard curve of iron(II) sulfate’s concentration versus absorbance was plotted. The FRAP activity was expressed from the curve equation as x 10^−4^ milligram Fe^2+^ equivalent per gram dry weight of extract (x 10^−4^ mg Fe^2+^ g^−1^ extract).

### Statistical analysis

**Figure 3:**
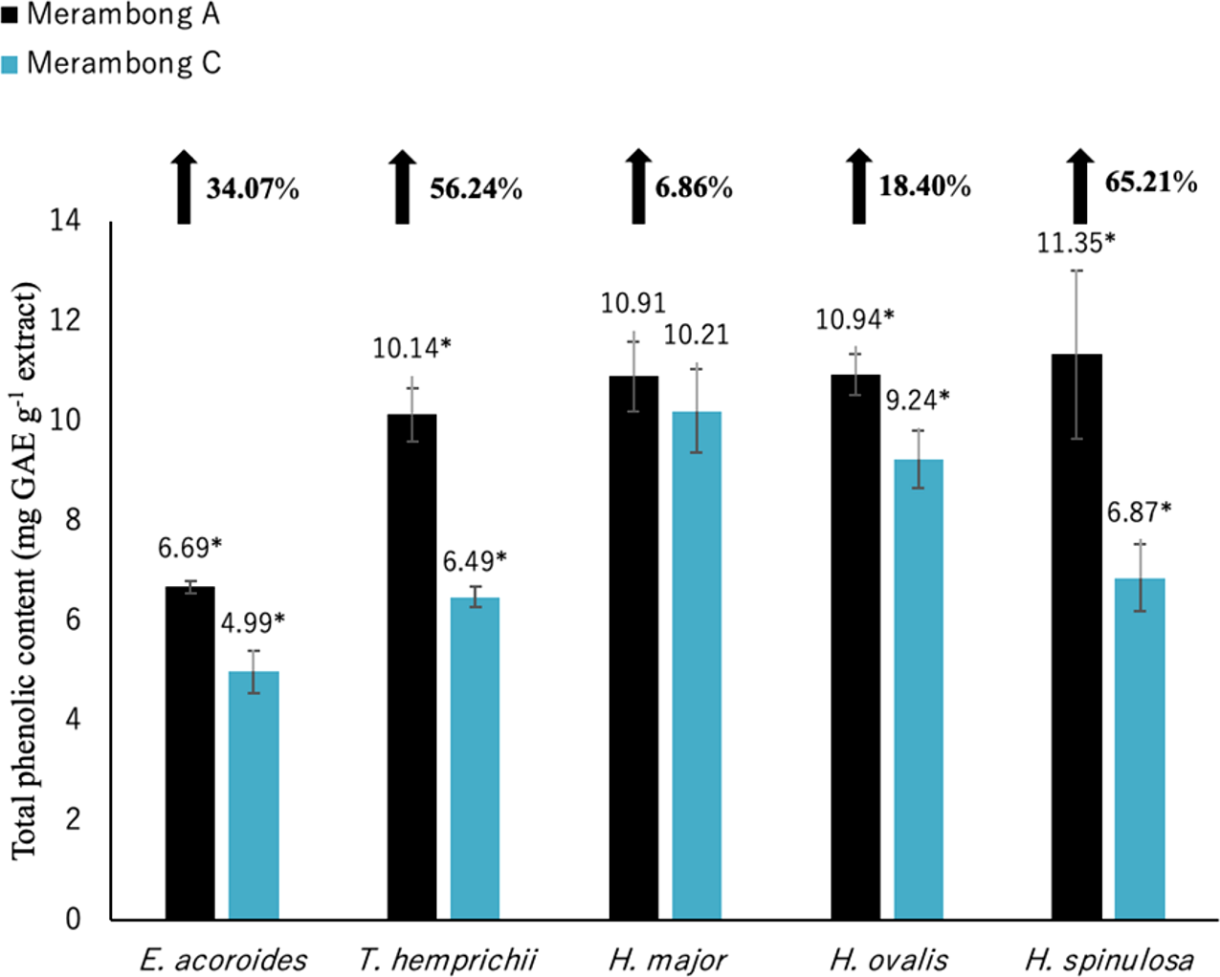
Total phenolic content of seagrasses from Merambong A and C shoals. Significantly higher mean value between studied sites was marked with an asterisk (*) (*t*-test, *p* < 0.05).

**Figure 4:**
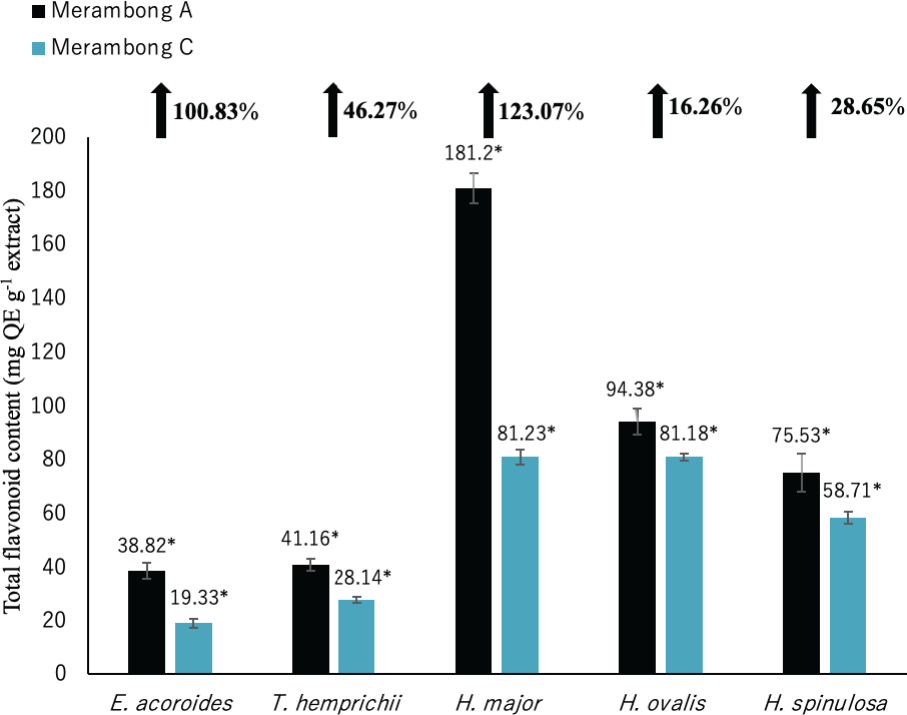
Total flavonoid content of seagrasses from Merambong A and C shoals. Significantly higher mean value between studied sites was marked with an asterisk (*) (*t*-test, *p* < 0.05).

**Figure 5:**
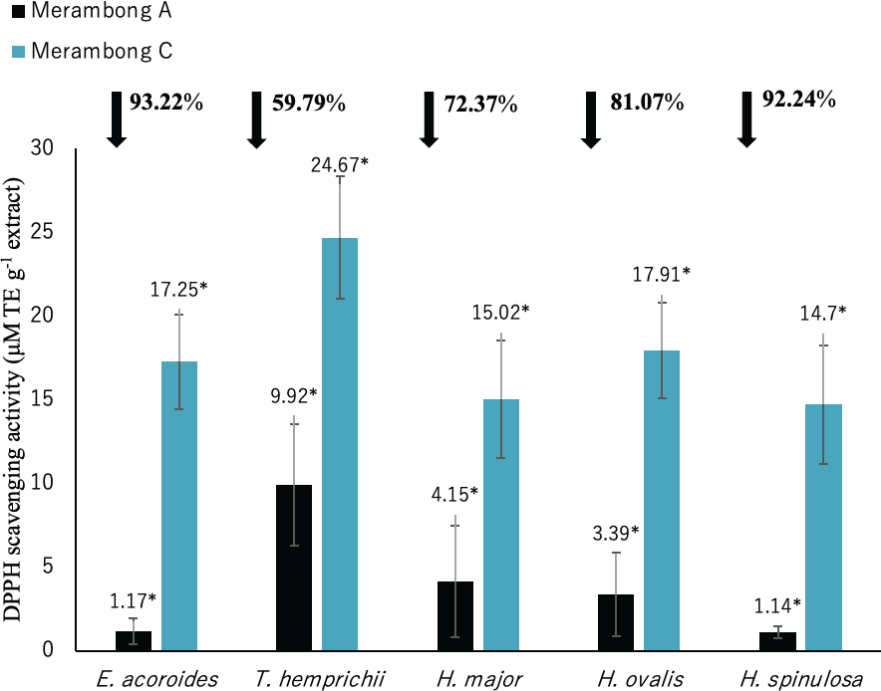
DPPH radical scavenging activity of seagrasses from Merambong A and C shoals. Significantly higher mean value between studied sites was marked with an asterisk (*) (*t*-test, *p* < 0.05).

**Figure 6:**
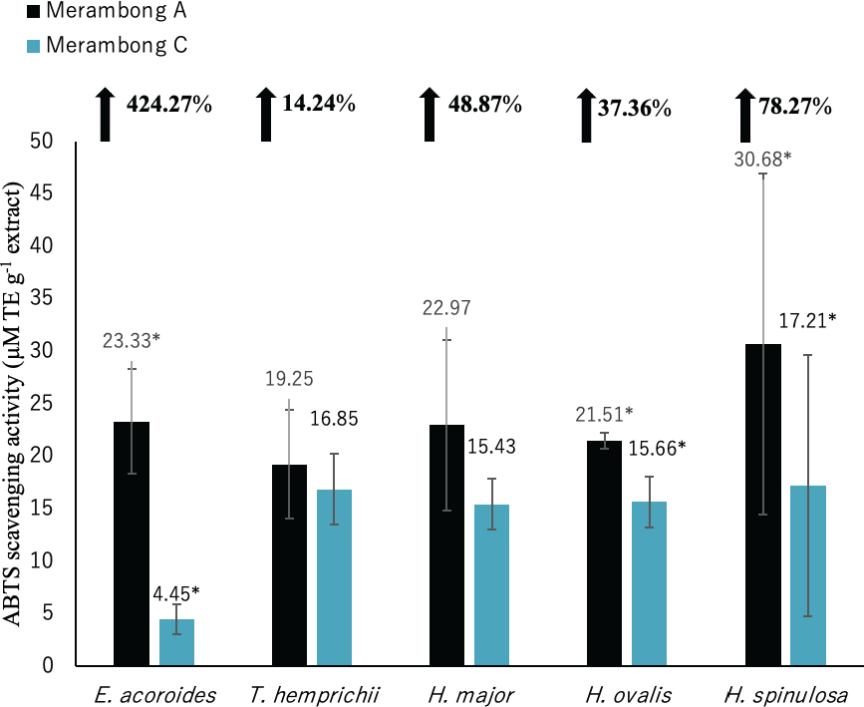
ABTS radical scavenging activity of seagrasses from Merambong A and C shoals. A significantly higher mean value between studied sites was marked with an asterisk (*) (*t*-test, *p* < 0.05).

**Figure 7:**
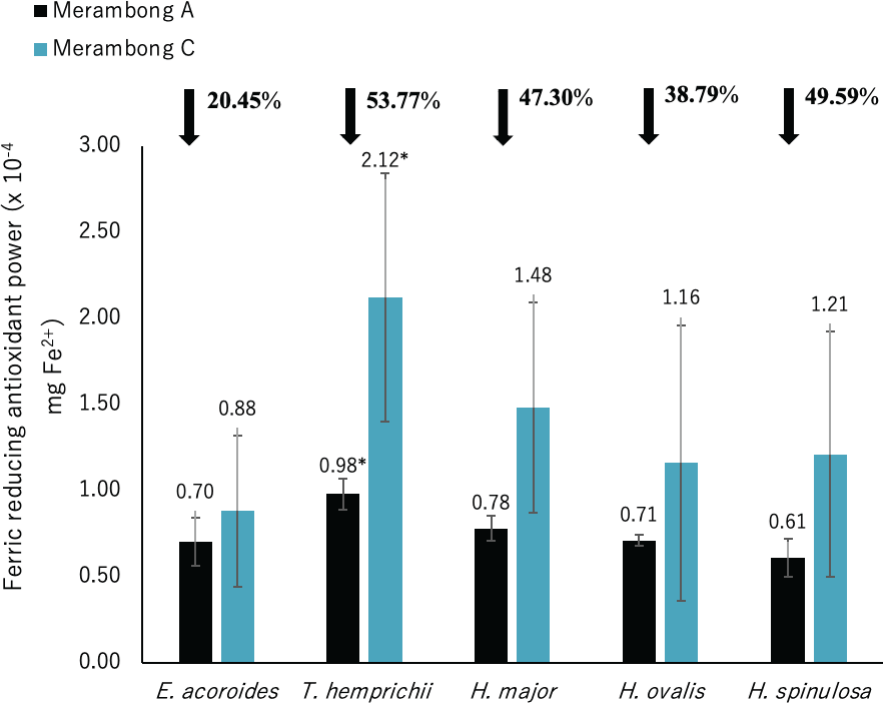
Ferric reducing antioxidant power (FRAP) of seagrasses from Merambong A and C shoals. Significantly higher mean value between studied sites was marked with an asterisk (*) (*t*-test, *p* < 0.05).

The results were reported as mean ±standard deviation. The water quality, seagrass morphological, and biochemical data were statistically analyzed using XLSTAT version 2014 software (Addinsoft, 2015). *T*-test (*p* < 0.05) was performed on water quality (Table 1), seagrass leaf dimension (Table 2), and seagrass biochemical (Figs. 3-7) from Merambong A and C, comparing seagrass of the same species. Pearson’s correlation analyses (*p* < 0.05) were performed between seagrass morphometric, water quality, and biochemical variables according to seagrass species, with the estimation of missing values (Tables 3-7). Significant Pearson correlation coefficients are presented in tabular form and interpreted as very high (>0.90), high (0.70 to 0.89), moderate (0.50 to 0.69), low (0.30 to 0.49), and negligible (<0.30) for positive correlation and vice versa (Hinkle, Wiersma & Jurs, 2003).

**Table 2:** Leaves dimensions are given as mean ± standard deviation of seagrasses from macroalgae colonized site (Merambong A) and non-colonized site (Merambong C) shoals.

**Leaves dimensions**	**MA**	**MC**	**Mean diff.**	**t**	**df**	**p**
***Enhalus acoroides***						
Leaf length (cm)	50.11 ± 13.14	45.15 ± 11.35	4.97	1.00	9	0.34
Leaf width (cm)	1.13 ± 0.14	0.85 ± 0.17	0.29	4.07	18	**0.001**
Leaf sheath length (cm)	15.45 ± 2.70	8.72 ± 0.55	6.73	4.88	6	**0.003**
Number of leaf per shoot	3.50 ± 0.58	3.25 ± 0.50	0.25	0.65	6	0.54
Leaf length to leaf width ratio	44.03 ± 9.19	53.10 ± 7.21	−9.07	−2.46	18	**0.02**
Leaf length to leaf sheath length ratio	3.87 ± 0.71	6.28 ± 1.11	−2.41	−3.65	6	**0.01**
***Thalassia hemprichii***						
Leaf length (cm)	13.58 ± 4.82	11.38 ± 2.95	2.20	1.51	28	0.14
Leaf width (cm)	0.57 ± 0.12	0.59 ± 0.09	−0.02	−0.59	28	0.56
Leaf sheath length (cm)	6.70 ± 1.33	5.32 ± 1.13	1.39	2.51	18	**0.02**
Number of leaf per shoot	3.00 ± 0.47	3.80 ± 0.63	−0.80	−3.21	18	**0.005**
Leaf length to leaf width ratio	25.05 ± 9.37	19.86 ± 6.36	5.19	1.78	28	0.09
Leaf length to leaf sheath length ratio	2.28 ± 0.40	2.39 ± 0.73	−0.11	−0.42	18	0.68
***Halophila major***						
Leaf length (mm)	29.72 ± 2.68	21.26 ± 2.66	8.46	19.55	150	**<0.0001**
Leaf width (mm)	15.21 ± 1.27	11.54 ± 1.37	3.66	17.10	150	**<0.0001**
Petiole length (mm)	26.84 ± 7.73	21.75 ± 4.66	5.09	4.92	150	**<0.0001**
Spaces between intra-marginal vein (mm)	0.40 ± 0.08	0.31 ± 0.04	0.09	8.95	150	**<0.0001**
Number of cross vein	24.62 ± 3.02	14.70 ± 1.57	9.92	25.39	150	**<0.0001**
Angle of cross vein (° )	60.07 ± 5.78	57.43 ± 4.80	2.63	3.07	150	**0.003**
Leaf length to leaf width ratio	1.96 ± 0.15	1.85 ± 0.16	0.11	4.33	150	**<0.0001**
Leaf length to petiole length ratio	1.21 ± 0.39	1.03 ± 0.33	0.18	2.98	150	**0.003**
Half lamina width to intra-marginal vein ratio	19.75 ± 3.96	19.14 ± 3.41	0.61	1.02	150	0.31
***Halophila ovalis***						
Leaf length (mm)	18.62 ± 2.17	17.06 ± 2.01	1.56	4.24	128	**<0.0001**
Leaf width (mm)	8.95 ± 1.11	7.67 ± 1.14	1.27	6.45	128	**<0.0001**
Petiole length (mm)	25.91 ± 6.83	22.17 ± 6.13	3.74	3.29	128	**0.001**
Spaces between intra-marginal vein (mm)	0.31 ± 0.04	0.26 ± 0.04	0.05	6.89	128	**<0.0001**
Number of cross vein	14.69 ± 1.50	14.77 ± 1.51	−0.08	−0.29	128	0.77
Angle of cross vein (° )	55.92 ± 5.92	60.74 ± 6.12	−4.82	−4.56	128	**<0.0001**
Leaf length to leaf width ratio	2.09 ± 0.22	2.24 ± 0.20	−0.15	−4.03	128	**<0.0001**
Leaf length to petiole length ratio	0.76 ± 0.20	0.85 ± 0.33	−0.08	−1.72	128	0.09
Half lamina width to intra-marginal vein ratio	14.71 ± 2.32	15.24 ± 3.22	−0.54	−1.09	128	0.28
***Halophila spinulosa***						
Leaf length (mm)	87.85 ± 24.19	84.49 ± 18.70	3.37	0.38	22	0.71
Leaf width (mm)	30.32 ± 1.49	28.86 ± 3.95	1.47	1.20	22	0.24
Leaflet length (mm)	15.80 ± 1.07	14.72 ± 1.99	1.07	4.51	178	**<0.0001**
Leaflet width (mm)	3.75 ± 0.35	3.38 ± 0.66	0.37	4.77	178	**<0.0001**
Number of leaflet	16.50 ± 4.44	16.83 ± 3.35	−0.33	−0.21	22	0.84
Leaf length to leaf width ratio	2.89 ± 0.79	2.97 ± 0.78	−0.08	−0.24	22	0.81
Leaflet length to leaflet width ratio	4.23 ± 0.36	4.43 ± 0.50	−0.19	−2.96	178	**0.003**

**Notes.**

Mean ± standard deviation in a row with bold *p* <  0.05 is significantly different (*t*-test). MA: Merambong A shoal and MC: Merambong C shoal.

## Results

### *In-situ* observation and water quality

*Ulva reticulata* was found attached to the seagrasses or free-floating and had formed a thick (5–30 cm) dense layer of extensive mat covering the seagrasses at Merambong A (MA Fig. 2A). Meanwhile, Merambong C (MC, Fig. 2C) was not affected by the macroalgae, serving as the non-colonized site. The man-made sand-filled embankment at Merambong B (MB, Fig. 2B) on top of Merambong shoal has blocked the natural water current flow and is susceptible to wave erosion, causing re-suspension sediments in the water column and the water to become turbid. High total suspended solids (TSS) were recorded at both MA and MC with the values of 102.33 ± 7.43 mg L^−1^ and 91.60 ± 14.59 mg L^−1^, respectively, in July 2014, along with the ongoing land reclamation activity (Table 1). No significant (*p* > 0.05) changes were recorded for the water quality parameters, *i.e*., temperature, atmospheric pressure, dissolved oxygen, conductivity, total dissolved solids, salinity, and pH, between MA and MC. However, only the water ammonia and nitrate showed significant (*p* < 0.05) differences between MA and MC. MA recorded significantly higher (*p* < 0.05) ammonia (0.06 ± 0.02 mg L^−1^) and nitrate (1.88 ± 0.73 mg L^−1^) concentrations compared to MC (0.03 ± 0.02 mg L^−1^ and 0.97 ±0.50 mg L^−1^, respectively). In MA, the macroalgae had densely covered the seagrasses. However, seagrass such as *E. acoroides* was still noticeable above the macroalgae mat since they can form a higher leaf canopy than other seagrass species (Fig. 2D). Muddy substrate was formed in MA, releasing a strong rotten egg odour when disturbed, and this is contradicted to the sandy substrate in MC (Fig. 2E). Patches of empty substrate were observed throughout MA shoal, and the seagrasses were observed to recover very slowly. Meanwhile, in MC, seagrasses grew abundantly and recovered quickly from the land reclamation activities (Figs. 2F and 2G).

### Morphological observation

Most of the seagrasses morphological parameters varied between *U. reticulata*-colonized site (MA) and non-colonized site (MC) shows in Table 2. The data were further analyzed for correlation of seagrasses with the water quality parameters and the finding are presented in Tables 3–7. *Enhalus acoroides* grown in macroalgae colonized site (MA) had significantly (*p* < 0.05) wider leaf width (1.13 ±0.14 cm) and longer leaf sheath length (15.45 ± 2.70 cm) as compared to the non-colonized site (MC). A moderate positive correlation was found between leaf sheath length and TSS ( *r* = 0.54, Table 3). While a moderate negative correlation was found between leaf sheath length and secchi disc depth (*r* = −0.62, Table 3). This is shows that when the TSS value is higher, the visibility decreases and caused the leaf sheath of *E. acoroide* s grow longer for adaptation to *U. reticulata*-colonized condition. Similarly, *T. hemprichii* grown at MA also possessed longer leaf sheath length (6.70 ± 1.33 cm) and a higher number of the leaf (3.00 ± 0.47 leaves per shoot) (*p* < 0.05, Table 2). Ammonia has a strong negative correlation with number of leaf per shoot (*r* =  − 0.72, *p* < 0.05) and positive moderate correlation with leaf sheath length (*r* = 0.55, *p* < 0.05, Table 4).

**Table 3:** Pearson correlation coefficient values between the variables of morphometric, water quality and biochemical of *Enhalus acoroides*.

**Variables**	**LSL**	**NOL**	**TSS**	**VSD**	**NH**_3_	**NO** ${}_{3}^{-}$	**NO** ${}_{2}^{-}$	**PO** ${}_{4}^{3-}$	**TPC**	**TFC**	**DPPH**	**ABTS**	**FRAP**
**LSL**	**1.00**												
**NOL**	**0.50**	**1.00**											
**TSS**	**0.54**	−0.27	**1.00**										
**VSD**	**−0.62**	0.04	**−0.71**	**1.00**							
**NH**_**3**_	0.44	0.37	0.15	0.12	**1.00**								
**NO**_**3**_ **-**	0.48	**0.59**	−0.01	0.18	**0.61**	**1.00**							
**NO**_**2**_ **-**	0.23	−0.30	0.11	−0.14	0.11	−0.10	**1.00**						
**PO** ${}_{\mathbf{4}}^{3-}$	0.10	0.26	−0.23	−0.07	0.02	0.26	−0.16	**1.00**					
**TPC**	**0.86**	0.18	**0.76**	**−0.76**	0.39	0.36	0.26	0.06	**1.00**			
**TFC**	**0.88**	0.29	**0.68**	**−0.72**	0.45	0.45	0.12	0.26	**0.96**	**1.00**		
**DPPH**	**−0.91**	−0.14	**−0.72**	**0.74**	−0.42	−0.37	−0.33	−0.09	**−0.95**	**−0.95**	**1.00**
**ABTS**	**0.93**	0.31	**0.60**	**−0.69**	0.39	0.37	0.25	0.22	**0.92**	**0.94**	**−0.95**	**1.00**
**FRAP**	−0.41	0.24	**−0.64**	0.41	−0.08	0.05	−0.26	0.35	−0.31	−0.26	0.43	−0.26	**1.00**

**Notes.**

Values in bold are different from 0 with a significance level alpha *p* < 0.05.

LSL leaf sheath length NOL number of leaf TSS total suspended solids VSD Visibility Secchi disc depth NH_3_ ammonia NO_3_^−^ nitrate NO_2_^−^ nitrite PO_4_^3−^ phosphate TPC total phenolic content TFC total flavonoid content DPPH DPPH radical scavenging activity ABTS ABTS radical scavenging activity FRAP ferric reducing antioxidant power

**Table 4:** Pearson correlation coefficient values between the variables of morphometric, water quality and biochemical of *Thalassia hemprichii*.

**Variables**	**LSL**	**NOL**	**TSS**	**VSD**	**NH**_**3**_	**NO** ${}_{\mathbf{3}}^{-}$	**NO** ${}_{\mathbf{2}}^{-}$	**PO** ${}_{\mathbf{4}}^{3-}$	**TPC**	**TFC**	**DPPH**	**ABTS**	**FRAP**
**LSL**	**1.00**												
**NOL**	−0.44	**1.00**											
**TSS**	0.33	−0.26	**1.00**										
**VSD**	−0.35	0.05	**−0.71**	**1.00**								
**NH**_**3**_	**0.55**	**−0.72**	0.15	0.12	**1.00**							
**NO**_**3**_	−0.48	−0.35	−0.01	0.18	**0.61**	**1.00**						
**NO**_**2**_ **-**	0.07	−0.26	0.11	−0.14	0.11	−0.10	**1.00**						
**PO** ${}_{\mathbf{4}}^{3-}$	0.38	0.15	−0.23	−0.07	0.02	0.26	−0.16	**1.00**					
**TPC**	**0.64**	−0.36	**0.59**	**−0.67**	0.49	0.49	0.20	0.16	**1.00**			
**TFC**	**0.65**	−0.29	**0.71**	**−0.73**	0.41	0.31	0.30	0.12	**0.94**	**1.00**		
**DPPH**	**−0.58**	0.25	**−0.72**	**0.75**	−0.31	−0.28	−0.29	−0.20	**−0.92**	**−0.94**	**1.00**
**ABTS**	−0.10	0.10	−0.16	0.00	0.03	0.05	**0.67**	−0.23	0.23	0.26	−0.21	**1.00**	
**FRAP**	**−0.75**	0.33	−0.45	0.49	**−0.51**	−0.27	−0.12	−0.18	**−0.73**	**−0.76**	**0.75**	−0.07	**1.00**

**Notes.**

Values in bold are different from 0 with a significance level alpha *p* < 0.05.

LSL leaf sheath length NOL number of leaf TSS total suspended solids VSD Visibility Secchi disc depth NH_3_ ammonia NO_3_^−^ nitrate NO_2_^−^ nitrite PO_4_^3−^ phosphate TPC total phenolic content TFC total flavonoid content DPPH DPPH radical scavenging activity ABTS ABTS radical scavenging activity FRAP ferric reducing antioxidant power

It was observed that *H. major* and *H. ovalis* were densely covered with *U. reticulata* in the MA site. These seagrass species showed high variation in terms of leaf dimensions between the studied sites. *Halophila major* and *H. ovalis* undercover of *U. reticulata* in MA, possessed significantly higher (*p* < 0.05) leaf dimension *i.e*., leaf length (29.72 ± 2.68 mm and 18.62 ± 2.17 mm, respectively), leaf width (15.21 ± 1.27 mm and 8.95 ± 1.11 mm, respectively), petiole length (26.84 ± 7.73 mm and 25.91 ± 6.83 mm, respectively), and spaces between intra-marginal vein (0.40 ± 0.08 mm and 0.31 ±0.04 mm, respectively) as shown in Table 2 and Figs. 2F & 2H. *Halophila spinulosa* had a longer leaflet length (15.80 ± 1.07 mm) and broader leaflet width (3.75 ± 0.35 mm), and lower leaflet length to leaflet width ratio value (4.23 ± 0.36 mm) in MA compared to MC site. Generally, no specific correlation was found between *Halophila’s* morphological dimension and water quality parameters (Tables 5–7).

**Table 5:** Pearson correlation coefficient values between the variables of morphometric, water quality and biochemical of *Halophila major*.

**Variables**	**CVA**	**TSS**	**VSD**	**NH**_**3**_	**NO** ${}_{\mathbf{3}}^{-}$	**NO** ${}_{\mathbf{2}}^{-}$	**PO** ${}_{\mathbf{4}}^{3-}$	**TPC**	**TFC**	**DPPH**	**ABTS**	**FRAP**
**CVA**	**1.00**											
**TSS**	0.12	**1.00**										
**VSD**	−0.12	**−0.71**	**1.00**								
**NH**_**3**_	0.19	0.15	0.12	**1.00**								
**NO**_**3**_ **–**	0.13	−0.01	0.18	**0.61**	**1.00**							
**NO**_**2**_ **–**	0.12	0.11	−0.14	0.11	−0.10	**1.00**						
**PO** ${}_{\mathbf{4}}^{3-}$	0.10	−0.23	−0.07	0.02	0.26	−0.16	**1.00**					
**TPC**	0.11	**0.54**	**−0.51**	−0.03	0.09	0.13	0.35	**1.00**			
**TFC**	0.22	**0.63**	**−0.69**	0.49	0.44	0.26	0.15	0.48	**1.00**		
**DPPH**	−0.19	**−0.71**	**0.74**	−0.26	−0.31	−0.29	−0.20	**−0.78**	**−0.90**	**1.00**	
**ABTS**	0.16	0.48	−0.45	0.44	**0.54**	0.19	−0.08	**0.51**	**0.67**	**−0.67**	**1.00**
**FRAP**	−0.14	**−0.53**	0.47	−0.52	−0.19	−0.04	0.07	−0.27	**−0.66**	**0.51**	**−0.50**	**1.00**

**Notes.**

Values in bold are different from 0 with a significance level alpha *p:* 0.05.

CVA cross vein angle TSS total suspended solids VSD Visibility Secchi disc depth NH_3_ ammonia NO_3_^−^ nitrate NO_2_^−^ nitrite PO_4_^3−^ phosphate TPC total phenolic content TFC total flavonoid content DPPH DPPH radical scavenging activity ABTS ABTS radical scavenging activity FRAP ferric reducing antioxidant power

### Biochemical properties

#### Total phenolic content (TPC)

Significantly higher (*p* < 0.05) TPC values were recorded in seagrasses grown in the site that colonized by *U. reticulata* (MA) except *H. major* compared to the non-colonized site (MC) as depicted in Fig. 3. *Halophila spinulosa* recorded the highest increment in TPC (65.21%) in *U. reticulata-* colonized site (MA), while *H. major* recorded the least increment of 6.86% (Fig. 3). Among the water quality parameter, TSS observed to have a moderate to strong positive correlation with TPC in all the studied species; *i e. E. acoroides* (*r* = 0.76, Table 3), *T. hemprichii* (*r* = 0.59, Table 4), *H. major* (*r* = 0.54, Table 5), *H. ovalis* (*r* = 0.64, Table 6) and *H. spinulosa* (*r* = 0.59, Table 7). In addition, a high negative correlation was found between TPC and secchi disc depth (visibility) for *E. acoroides* (*r* =  − 0.76, Table 3), and *H. ovalis* (*r* =  − 0.71, Table 6), while moderate negative correlation was observed in *T. hemprichii* ( *r* =  − 0.67, Table 4), *H. major* (*r* =  − 0.51, Table 5) and *H. spinulosa* (*r* =  − 0.59, Table 7).

**Table 6:** Pearson correlation coefficient values between the variables of morphometric, water quality and biochemical of *Halophila ovalis*.

**Variables**	**IV**	**TSS**	**VSD**	**NH**_**3**_	**NO** ${}_{\mathbf{3}}^{-}$	**NO** ${}_{\mathbf{2}}^{-}$	**PO** ${}_{\mathbf{4}}^{3-}$	**TPC**	**TFC**	**DPPH**	**ABTS**	**FRAP**
**IC**	**1.00**											
**TSS**	0.03	**1.00**										
**VSD**	−0.10	**−0.71**	**1.00**								
**NH**_**3**_	0.14	0.15	0.12	**1.00**								
**NO**_**3**_ **-**	0.19	−0.01	0.18	**0.61**	**1.00**							
**NO**_**2**_ **-**	0.08	0.11	−0.14	0.11	−0.10	**1.00**						
**PO** ${}_{\mathbf{4}}^{3-}$	0.20	−0.23	−0.07	0.02	0.26	−0.16	**1.00**					
**TPC**	0.20	**0.64**	**−0.71**	0.31	0.39	0.17	0.22	**1.00**				
**TFC**	0.23	**0.54**	**−0.66**	0.38	0.44	0.07	0.35	**0.93**	**1.00**		
**DPPH**	−0.21	**−0.74**	**0.76**	−0.36	−0.35	−0.28	−0.15	**−0.91**	**−0.93**	**1.00**
**ABTS**	0.20	**0.63**	**−0.66**	0.30	0.40	0.37	0.16	**0.84**	**0.78**	**−0.90**	**1.00**
**FRAP**	−0.10	−0.13	0.15	**−0.55**	−0.24	0.28	−0.04	−0.15	−0.35	0.26	0.03	**1.00**

**Notes.**

Values in bold are different from 0 with a significance level alpha *p* < 0.05.

IV spaces between intra-marginal vein TSS total suspended solids VSD Visibility Secchi disc depth NH_3_ ammonia NO_3_^−^ nitrate NO_2_^−^ nitrite PO_4_^3−^ phosphate TPC total phenolic content TFC total flavonoid content DPPH DPPH radical scavenging activity ABTS ABTS radical scavenging activity FRAP ferric reducing antioxidant power

#### Total flavonoid content (TFC)

Total flavonoid content for all seagrass species were significantly higher in the area colonized by *U. reticulata* (MA), compared to the non-colonized site (MC) in Fig. 4 (*t*-test, *p* <0.05). Under the *U. reticulata*-colonized site (MA), *H. major* recorded the highest increment of TFC with 123.07%, while *H. ovalis* recorded the lowest increment of 16.26% (Fig. 4). A high positive correlation was observed between seagrass TFC and TSS for *T. hemprichii* (*r* = 0.71, Table 4), while a moderate positive correlation for *E. acoroides* (*r* = 0.68, Table 3), *H. major* (*r* = 0.63, Table 5), *H. ovalis* (*r* = 0.54, Table 6) and *H. spinulosa* (*r* = 0.63, Table 7).

**Table 7:** Pearson correlation coefficient values between the variables of morphometric, water quality and biochemical of *Halophila spinulosa*.

**Variables**	**LL**	**NOL**	**TSS**	**VSD**	**NH**_**3**_	**NO** ${}_{\mathbf{3}}^{-}$	**NO** ${}_{\mathbf{2}}^{-}$	**PO** ${}_{\mathbf{4}}^{3-}$	**TPC**	**TFC**	**DPPH**	**ABTS**	**FRAP**
**LL**	**1.00**												
**NOL**	**0.89**	**1.00**											
**TSS**	0.30	0.40	**1.00**										
**VSD**	−0.34	−0.38	**−0.71**	**1.00**								
**NH**_**3**_	0.08	0.18	0.15	0.12	**1.00**								
**NO**_**3**_ **-**	0.14	0.25	−0.01	0.18	**0.61**	**1.00**							
**NO**_**2**_ **-**	0.08	−0.02	0.11	−0.14	0.11	−0.10	**1.00**						
**PO** ${}_{\mathbf{4}}^{3-}$	−0.25	−0.25	−0.23	−0.07	0.02	0.26	−0.16	**1.00**					
**TPC**	0.24	0.32	**0.59**	**−0.59**	0.48	0.44	0.25	0.08	**1.00**			
**TFC**	0.40	**0.50**	**0.63**	**−0.69**	0.37	0.35	0.16	0.18	**0.59**	**1.00**		
**DPPH**	−0.41	−0.49	**−0.76**	**0.74**	−0.40	−0.33	−0.35	−0.05	**−0.87**	**−0.85**	**1.00**
**ABTS**	0.29	0.33	0.33	−0.38	**0.52**	**0.51**	0.28	−0.01	**0.93**	0.40	**−0.72**	**1.00**
**FRAP**	−0.23	−0.31	−0.14	0.22	**−0.58**	−0.27	0.09	−0.13	−0.40	−0.49	0.46	−0.40	**1.00**

**Notes.**

Values in bold are different from 0 with a significance level alpha *p* = 0.05.

LL leaf length NOL number of standing leaf TSS total suspended solids VSD Visibility Secchi disc depth NH_3_ ammonia NO_3_^−^ nitrate NO_2_^−^ nitrite PO_4_^3−^ phosphate TPC total phenolic content TFC total flavonoid content DPPH DPPH radical scavenging activity ABTS ABTS radical scavenging activity FRAP ferric reducing antioxidant power

#### Antioxidant activities (DPPH, ABTS, and FRAP)

The lower value of DPPH shows the higher antioxidant activity. Significantly higher antioxidant activities was recorded in all seagrass species at MA with lower DPPH value as illustrated in Fig. 5 (*p* < 0.05). Reduced DPPH activity was recorded for seagrasses under *U. reticulata*-colonized site with *E. acoroides* recorded the highest reduction of 93.22%, while *T. hemprichii* recorded the least reduction of 59.79%.

DPPH shows high to very high negative correlation with TPC and TFC in all seagrass species (*p* < 0.05). *Enhalus acoroides* highly negative correlated with TPC and TFC at *r* =  − 0.95 (Table 3), and *T. hemprichii* (TPC *r* =  − 0.92, TFC *r* =  − 0.94, Table 4), *H. ovalis* (TPC *r* =  − 0.91, TFC *r* =  − 0.93, Table 6) and *H. major* (*r* =  − 0.90, Table 5). In addition, DPPH also showed high negative correlation with TPC for *H. major* (*r* =  − 0.78, Table 5), and TPC and TFC for *H. spinulosa* (TPC *r* =  − 0.87, TFC *r* =  − 0.85, Table 7).

Significantly higher ABTS activity was recorded for *E. acoroides*, *H. ovalis* and *H. spinulosa* in *U. reticulata*-colonized site (MA), compared to the non-colonized site (MC) (Fig. 6, *t*-test, *p* < 0.05). *Enhalus acoroides* recorded the highest increment in ABTS (424.27%) in *U. reticulata-* colonized site (MA), followed by *H. spinulosa* (72.87%) and *H. ovalis* (37.76%). A very high positive correlation was observed between ABTS activity and seagrass TPC and TFC of *E. acoroides* with *r* = 0.92*,* and *r* = 0.94 respectively (Table 3) and TPC *H. spinulosa* (*r* = 0.93*,* Table 7). While high positive correlation was also recorded between ABTS activity with TPC and TFC of *H. ovalis* (*r* = 0.84*, r* = 0.78, respectively, Table 6).

Significantly higher FRAP activity was only recorded for *T. hemprichii* in the non-colonized *U. reticulata* site (MC) compared to the colonized site (MA), as illustrated in Fig. 7 (*t*-test, *p* < 0.05). Reduced FRAP activity was recorded for seagrasses in the *U. reticulata*-colonized site, where *T. hemprichii* recorded the highest reduction of 53.77%, while *E. acoroides* recorded the lowest reduction of 20.45%. A high negative correlation was observed between *T. hemprichii*’s TPC and TFC with FRAP activity at *r* =  − 0.73 and *r* =  − 0.76 respectively (Table 4). FRAP activity in other seagrasses were not correlated with TPC and TFC content.

## Discussion

Comparison between Merambong shoals water quality and Malaysian Marine Water Quality Standards for Sensitive Marine Habitats include seagrass (Class 1) (MMWQS) has revealed that minimum and maximum values recorded for nitrate and phosphate concentrations in Merambong shoals are below the criteria set limit of 10.0 mg L^−1^ and 5.0 mg L^−1^ respectively (Department of Environment Malaysia, 2019) referring to Table 1 in the Results.

The seagrasses’ morphological responses may be attributed to a variety of environmental factors, including nutrient availability (Lee & Dunton, 2000; Ali et al., 2012; La Nafie et al., 2013), light availability (Collier, Waycott & Ospina, 2012; La Nafie et al., 2013; Martínez-Crego et al., 2016; Bertelli & Unsworth, 2018), hydrodynamics (Schanz & Asmus, 2003; Peralta et al., 2006) and sulfide intrusion (Terrados et al., 1999; Kilminster et al., 2008).

The high nutrient concentrations may have been derived from anthropogenic inputs such as industrial and domestic wastewater and agricultural runoff along the upstream rivers of Sungai Pulai estuary (Ahmad et al., 2014). These inputs provide a continuous supply of excessive nitrogen (N) and phosphorus (P) into the rivers, which can contribute to algae bloom, anoxic water, and acidification of the marine ecosystem (Ngatia et al., 2019).

Both sites recorded eutrophic water columns with minimum and maximum nitrate and phosphate concentrations, below the criteria set limit of 10.0 mg/L and 5.0 mg/L respectively, MMWQS (Class 1) recommended limit (Department of Environment Malaysia, 2019). Seasonal bloom of several macroalgae, *i.e*., *U. reticulata*, *Amphiroa fragilissima,* and *Hydropuntia edulis,* was observed at the shoal during June to August 2013, which have coincided with the Southwest monsoon season (Muta Harah et al., 2014). Yet, the altered shoal disrupted their seasonal growing pattern, and *U. reticulata* was found to be the only dominant species in the shoal in July 2014. The sheltered and eutrophic waters in MA provide favourable conditions for the proliferation of *U. reticulata*, and outcompetes the slower-growing seagrasses in MA. The sheltered seagrass bed colonized with *U. reticulata* in MA has caused greater seagrass loss and habitat fragmentation. The acclimatized Hydrocharitaceae seagrass in MA produced larger leaf dimensions as compared to MC. Among the studied species, simple broad-leaved *H. major* and *H. ovalis* in MA showed significant increments in the morphometry variables, *i.e*., leaf length, leaf width, petiole length, and space between intra-marginal veins, which is in accordance to the previous findings in *Halophila* species have high leaf morphometry variation across geographic locations and are highly sensitive to various environmental disturbances (Annaletchumy et al., 2005; Nguyen et al., 2014).

Similar to other studies, green tides of *U. reticulata* were also seen in eutrophic waters in several tropical regions, such as Thailand (Buapet et al., 2008), Philippines (Largo et al., 2004), and China (Wu et al., 2018). *Ulva* species is a fast-growing opportunistic macroalgae and has higher nitrogen and phosphate uptake rates than other species (Luo, Liu & Xu, 2012; Liu et al., 2013). This fact is in line with the present findings where significantly higher (*p* < 0.05) ammonia (0.06 ± 0.02 mg L^−1^) and nitrate (1.90 ± 0.70 mg L^−1^) concentrations were recorded in MA than MC. This caused the primary producer of the meadow to shift from seagrass to the *U. reticulata*. A previous study has also shown a faster growth rate of *U. reticulata* when cultured under higher nitrate water (Buapet et al., 2008). An increase in nitrogen supply raises the N uptake rate, improves photosynthetic performance, and accelerates the growth of the *Ulva* sp. that can eventually occupy the entire seagrass meadow (Valiela et al., 1997).

The active uptake systems influence seagrass growth for nitrate (NO^3−^), phosphate (PO_4_^3−^), and ammonium (NH_4_^+^) from sediment pore water and water column (Touchette & Burkholder, 2000). Short-term nutrient enrichment in oligotrophic water increases the seagrass shoot biomass and density, whereas a later long-term exposure as the water becomes eutrophic, the water condition will inhibit the seagrass growth (Cabaço et al., 2013). However, our findings contradict to Touchette, Burkholder & Glasgow (2003) and Cabaço et al. (2013), who had reported significant growth reduction after long-term exposure to nutrient enrichment. MA suffered from seagrasses loss with noticeable seagrass-devoid patches throughout the shoal, and yet the thrived seagrass in MA produced significantly larger leaf dimensions. The loss is possibly related to the prolonged nitrogen fertilization of ammonia and nitrate (Sandoval-Gil et al., 2019). It becomes aggravated in a low water exchange seagrass bed, resulting in a rapid decline of the seagrass cover (Burkholder, Mason & Glasgow Jr, 1992). Seagrasses loss in MA disrupted the seagrasses below-ground rhizome network and caused clonal integration.

Moreover, the construction of the sand-filled embankment (MB) has changed the hydrodynamic forces in both Merambong A (MA) and Merambong C (MC), where MA is sheltered from strong wind-blown waves and tidal currents, while MC is continuously exposed to these parameters. Tidal hydrodynamics influence the nutrient flow, sedimentation rate, and physical stability of the seagrass beds (Lanuru et al., 2018). Increasing wave force elevates the degree of physical stability but reduces the nutrient availability in the seagrass bed sediment (El-Hacen et al., 2019). Changes in hydrodynamic regime combined with eutrophic waters may have affected the macrophytes composition at the Merambong shoal. The hydrodynamics in Merambong shoal is naturally regulated by the strong tidal and wind-blown waves from the open sea into the Johor Strait. However, the constructed MB had sheltered the seagrasses and promoted *U. reticulata*, potentially reducing the hydrodynamic forces in MA. MC, on the other hand, was continuously affected by these parameters. As a result, seagrasses in MA had more significant leaf dimensions than MC, possibly due to the reduced water current velocity as suggested by Schanz & Asmus (2003) and Peralta et al. (2006). According to Schanz & Asmus (2003) and El-Hacen et al. (2019), the seagrasses are constantly affected by hydrodynamic forces, *i.e*., strong tidal currents and waves, which directly affect seagrass morphology and physiology. Additionally, because sediment is deposited on seagrass beds due to their ability to decrease current velocities and weaken wave energy (Gambi, Nowell & Jumars, 1990), seagrass presence may enhance macroalgae growth, especially *U. reticulata* on the soft sediments Buapet et al. (2008).

Nutrient enrichment had its primary effects on seagrass, but it has also encouraged the overgrowth of macroalgal associated with light reduction (Touchette & Burkholder, 2000; Schmidt et al., 2012; Han & Liu, 2014). In MA, colonization of *U. reticulata* has formed a thick and dense layer of the macroalgae in the area, which reduced the light passing through to the seagrass underneath and subsequently decreased the seagrass cover. This observation is similar to the reported decline of temperate seagrass *Posidonia oceanica* meadows when colonized with *Caulerpa* sp. (Terrados et al., 1999; Garcias-Bonet et al., 2008). Light is a fundamental driving factor for sustaining primary productivity of photosynthetic organisms, and seagrasses require at least 6 to 10 mol photon m^−2^d^−1^ of daily light to maintain leaf growth (Collier et al., 2016). In general, light deprivation decreases survival rate (Longstaff & Dennison, 1999), growth rate (Longstaff & Dennison, 1999; Collier, Waycott & Ospina, 2012; Collier et al., 2016), shoot density (Longstaff & Dennison, 1999; Collier et al., 2007; Collier et al., 2016; Suykerbuyk et al., 2018), and biomass (Longstaff & Dennison, 1999; Collier et al., 2007; Suykerbuyk et al., 2018) of seagrasses. These facts have supported the present findings where the colonization of *U. reticulata,* associated with reduced light exposure in MA, would negatively impact the seagrass survival and the growth. Areas densely covered with *U. reticulata* in MA-induced seagrasses died off and led to the formation of patches of the seagrass-devoid substrate on the shoal. The plant death is possibly due to their inability to maintain a positive carbon balance (Ralph et al., 2007) and regulate phytotoxic end-product accumulation (Longstaff et al., 1999) under low light conditions. Plant death due to light deprivation for *Halophila* species is notably faster than other seagrasses (Longstaff et al., 1999). It is expected then that the prolonged light-limiting condition in MA could lead to mass mortality of *H. major*, *H. ovalis,* and *H. spinulosa*. However, similar to our findings, the rapid growth and short life cycle of *Halophila* species reflect the species’ strong recovery capacity (Longstaff & Dennison, 1999; Cabaço, Santos & Duarte, 2008).

Additionally, the seagrasses loss in MA disrupted the seagrasses below-ground rhizome network and caused clonal integration. Similar to our study, Tuya et al. (2013) has observed that the excessive water fertilization on seagrass *Cymodocea nodosa* with isolated clonal connection has significantly reduced its horizontal growth and productivity but produced higher shoot density, leaf per shoot, leaf surface, and biomass as compared to those without clonal integration. The enhanced morphological features may be linked to the higher assimilation of P in above-ground and N in below-ground tissues of the isolated clonal (Tuya et al., 2013). While *U. reticulata* has a short life cycle and proliferates rapidly in higher nitrate water, these characteristics also induce rapid degradation, bringing mass decomposition of the macroalgae (Buapet et al., 2008). Ephimeral macroalgae such as *Ulva* sp. have a low C:N ratio and less structural material than rooted macrophytes, enabling them to be readily degraded by bacteria (Duarte, 1995). Decomposition of the macroalgae releases organic carbon and nutrient inputs into the sediment, stimulating the production of sediment sulfide from the sulfate-reducing activity of the prokaryotes (Terrados et al., 1999; Ugarelli et al., 2017). This resulted in the black sedimentation with strong rotten egg odour in macroalgae colonized shoal of MA. The pungent odour is due to the high sediment sulfide concentration, inhibits seagrass meristematic activity, and even causes seagrass die-off (Terrados et al., 1999; Garcias-Bonet et al., 2008; Ugarelli et al., 2017). Sulfide tolerance concentration also varies according to seagrass species, as low as 10 µM for *P. oceanica* (Calleja, Marbà & Duarte, 2007) to as high as 750 µM for *Thalassia testudinum* (Borum et al., 2005). This is in agreement with the present findings on the decline of seagrasses species in MA shoal and is also supported by Kilminster et al. (2008), where sediment enriched with sodium sulfide (1.1–4.2 g m^−2^) significantly reduces *H. ovalis* growth rate, biomass, and internode length.

Generally, seagrasses are rich in secondary metabolites (phenolic compounds), including phenolic acids (Zapata & McMillan, 1979; Grignon-Dubois et al., 2012), flavonoids (Cannac et al., 2006; Bitam et al., 2010), condensed tannins (McMillan, 1984) and lignin (Klap, Hemminga & Boon, 2000). These metabolites are essential in the building up of resistance against survival in a harsh environment. It is therefore conceivable that the result of this study showed that the higher phytochemical properties among the seagrasses grown under macroalgae colonized shoal (MA) in comparison to non-colonized shoal (MC). Several studies have linked to the production of secondary metabolites in seagrasses which is strongly dependent on various environmental factors such as hydrodynamics (Grignon-Dubois et al., 2012), nutrient availability (VanKatwijk et al., 1997; Cannac et al., 2006; Martínez-Crego et al., 2016; Kajela & Mvungi, 2018), intraspecific competition between macroalgae (Cuny et al., 1995; Agostini, Desjobert & Pergent, 1998; Ferrat, Fernandez & Dumay, 2001; Dumay et al., 2004), light availability (Vergeer, Aarts & De Groot, 1995; Sneed, 2005; Gavin & Durako, 2012; Silva et al., 2013) and heavy metal contamination (Li et al., 2012; Ferrat et al., 2012). Numerous studies have explained that seagrasses tend to produce more or higher levels of bioactive compounds and antioxidants as a preventive or protective measure against oxidative and abiotic stress. According to Grignon-Dubois et al. (2012)*,* the *Zostera noltii* that grown on intertidal seagrass beds (Arcachon Lagoon and Cadiz Bay) with extreme tidal currents and emersion/submersion cycles, exhibited higher phenolic acid production, *i.e.,* rosmarinic, caffeic and zosteric acids, as compared to those at sheltered subtidal bay (Nitja Bay and Alfacs Bay). This is contradicting the finding by Gavin & Durako (2011), where the flavonoid content of *Halophila johnsonii* was not significantly different between intertidal and subtidal, probably due to the proximity of the sampling sites. Moreover, the synthesis of secondary metabolites in seagrasses also depends on the availability of N and P in the water column and pore water (Buchsbaum, Short & Cheney, 1990). Seagrass allocates more carbohydrates for growth when under adequate nutrient conditions but focuses on the production of secondary metabolites when under nutrient stress conditions (Kajela & Mvungi, 2018). However, extreme nutrient enrichment can significantly inhibit the production of secondary metabolites in seagrass and even causes plant death (VanKatwijk et al., 1997). An inverse correlation between water nutrients (NO^3−^ and PO_4_^3−^) and phenolic content was reported for seagrass *T. hemprichii* (Kajela & Mvungi, 2018). High ammonium (125 µM) treatment decreases the phenolic content and induces necrosis in *Z. marina* after five weeks (VanKatwijk et al., 1997). *Zostera noltii* meadow enriched with water nutrient (44 µM NO^3−^, 3.9 µM PO_4_^3−^ and 74 µM NH_4_^+^) also showed a significant reduction of its phenolic content (Martínez-Crego et al., 2016).

The overwhelming growth of *U. reticulata* in MA has shown intraspecific competition between the macroalgae and Hydrocharitaceae seagrass. Previous studies showed that the colonization of macroalgae *Caulerpa taxifolia* on seagrass meadow increases the total water-soluble phenolic content in *P. oceanica* (Cuny et al., 1995; Ferrat, Fernandez & Dumay, 2001). However, the production of phenolics as an effect of intraspecific competition between *Caulerpa taxifolia* and *P. oceanica* is lower than those affected by herbivores’ over-grazing, nutrient enrichment, heavy metal contamination, and interspecific competition between seagrass (Agostini, Desjobert & Pergent, 1998). The seagrass phenolics production also appears to be at a species-specific interaction level, with significant increment observed in *P. oceanica* colonized only by *C. taxifolia*, but not for *P. oceanica* occupied by *C. racemosa* (Dumay et al., 2004). In our case, the increment of phenolics content (TPC and TFC) is probably due to the competition for light. The *U. reticulata* mass can form as a free-floating dense mat above seagrass reducing the light availability for seagrass underneath. Light properties, including intensity, duration, and wavelength, can affect the production of phenolic in plants (Idris et al., 2018). Some seagrasses had shown signs of photo-acclimation under light-deprived conditions (Silva et al., 2013) and responded physiologically according to their minimum light requirements (Collier et al., 2016). For example, light shading (75–228 µmol photon m^−2^ s^−1^) treatment increases the phenolic content of *Z. marina*, but not for *C. nodosa* (Silva et al., 2013). This is because *C. nodosa* is more resilient to light deprivation and can accumulate higher starch and soluble sugar in its rhizome than the *Z. marina* (Silva et al., 2013). Total flavonoid content was also higher in the shaded *H. johnsonii,* and the flavonoids were significantly correlated with the ABTS antioxidant activity (Gavin & Durako, 2012). On the other hand, some seagrasses such as *Z. marina* (Vergeer, Aarts & De Groot, 1995) and *T. testudinum* (Sneed, 2005) produce a reported higher level of phenolic content when grown under high light intensity. Nevertheless, allelopathic interaction between macroalgae and seagrass is still under study and could be considered a possible factor affecting the production of the phenolic in the seagrasses. A high level of metal contamination was reported at Sungai Pulai estuary and had accumulated in the seagrass tissues (Ahmad et al., 2015). Trace metals can affect the seagrass secondary metabolites production depending on the metal type and seagrass’ tolerance capacity (Li et al., 2012).

Present findings revealed that a strong and positive correlation was between metabolites content (TPC and TFC) and antioxidant activities (ABTS) and strong and negative correlation (DPPH and FRAP) in all the studied seagrass species. Numerous studies cited in the literature showed a similar pattern of strong correlation (Kannan, Arumugam & Anantharaman, 2010). This suggests that TPC is the main contributor to the antioxidant activity in seagrass species. A strong positive correlation pattern between TPC and ABTS was also observed in temperate seagrasses from the Hydrocharitaceae family, *i.e*., *Halophila stipulacea* and *H. ovalis* and Cymodoceaceae family *Thalassodendron ciliatum* during the winter season with short daylight and low temperature (Ramah et al., 2014). In general, while antioxidant substances, *i.e*., phenolic compounds, can prevent oxidation processes by donating an electron to the free radicals, the reaction also results in the formation of non-reactive antioxidants and other by-products accumulating in plant tissues (Metodiewa et al., 1999), which may explain the high metabolites (TPC and TFC) content with high DPPH and FRAP activities for seagrasses in MA.

## Conclusion

The present study demonstrated the responses of seagrasses from the family of Hydrocharitaceae (*E. acoroides, T. hemprichii, H. major, H. ovalis,* and *H, spinulosa*) under the colonization of macroalgae *U, reticulata* in the reclaimed shoal. Seagrass leaves’ morphological variations were observed between Merambong A (*U. reticulata*-colonized site) and Merambong C (non-colonized site) suggested that species of Hydrocharitaceae family undergo leaf morphometric changes to adapt to the altered environment. Furthermore, all seagrass species in *U. reticulata*-colonized sites possessed higher total phenolic and flavonoid contents than those of the non-colonized sites shows that the plants exhibited higher levels of bioactive compounds as a preventive or protective measure against oxidative and abiotic stress. The increment of seagrasses’ phenolic compounds in Merambong A shoal was most probably due to the shading effect from *U. reticulata* mass, forming thick mats above the seagrass beds that reduce the light availability. Although seagrasses from Merambong A contained high polyphenols, especially flavonoids, the seagrasses’ antioxidant activities, such as DPPH and FRAP, were significantly lower compared to those of Merambong C. This indicates that different metabolites were produced under different environment conditions. It is necessary to research the synergistic effect of anthropogenic nutrient loads on the interaction between seagrasses and macroalgae in eutrophic areas, which can provide valuable information to decrease the adverse impact of macroalgae bloom on seagrasses.

##  Supplemental Information

10.7717/peerj.12821/supp-1 Supplemental Information 1 Raw data for morphological and phytochemical analysis

10.7717/peerj.12821/supp-2 Supplemental Information 2 GPS coordinates for the seagrass species collected in Merambong A (MA) and Merambong C (MC)

## Additional Information and Declarations

### Data Availability

The following information was supplied regarding data availability: The raw data is available in the Supplementary Files and at GitHub: https://github.com/emmclan/D1-DATA-FOR-PEERJ/commit/0971d74670ad32cade57dc5201923c3b52e10a14.
